# Linagliptin inhibits lipopolysaccharide-induced inflammation in human U937 monocytes

**DOI:** 10.1186/s41232-018-0071-z

**Published:** 2018-08-20

**Authors:** Shiho Yamadera, Yuya Nakamura, Masahiro Inagaki, Sachiyo Kenmotsu, Tetsuhito Nohara, Naoki Sato, Tatsunori Oguchi, Mayumi Tsuji, Isao Ohsawa, Hiromichi Gotoh, Yoshikazu Goto, Akihiko Yura, Yuji Kiuchi, Shinichi Iwai

**Affiliations:** 1 Department of Healthcare and Regulatory Sciences, Showa University School of Pharmacy, Shinagawa-ku, Tokyo Japan; 2 Department of Pharmacology, Showa University School of Medicine, 1-5-8 Hatanodai, Shinagawa-ku, Tokyo Japan; 3 Saiyu Soka Hospital, Soka City, Saitama-ken Japan; 4 Department of Chemistry, College of Arts and Sciences, Showa University, Fujiyoshida City, Yamanashi-ken Japan; 5 School of Medicine, Showa University Preventive Medicine center, Koto-ku, Tokyo Japan

**Keywords:** Anti-inflammatory effects, Linagliptin, Human U937 monocytes, Interleukin 6, Lipopolysaccharide

## Abstract

**Background:**

Atherosclerosis and inflammation are more common in patients with diabetes than in patients without diabetes, and atherosclerosis progression contributes to inflammation. Therefore, anti-inflammatory therapy is important for the prognosis of patients with diabetes. Linagliptin is the only bile-excreted, anti-diabetic oral dipeptidyl peptidase-4 (DPP-4) inhibitor. Although the anti-inflammatory effects of DPP-4 inhibitors in vivo and in vitro have been reported, few in vitro studies have examined the effects of linagliptin using monocytes, which play a central role in arteriosclerosis-related inflammation. Herein, we assessed the anti-inflammatory effects of linagliptin in human U937 monocytes.

**Methods:**

U937 cells at densities of 1 × 10^6^ cells/mL were cultured in Roswell Park Memorial Institute medium supplied with 10% fetal bovine serum and treated with 100 nM phorbol myristate acetate for 48 h for differentiation into macrophages. The media were replaced, and the cells were pretreated with 1, 5, 10, 50, and 100 nM linagliptin for 1 h or were left untreated. The media were then replaced again, and the cells were treated with 1 μg/mL lipopolysaccharide (LPS) or 10 nM interleukin (IL)-1β only, in combination with 1, 5, 10, 50, and 100 nM linagliptin or were left untreated. The extracted media were used to measure IL-6 and tumor necrosis factor (TNF)-α levels using enzyme-linked immunosorbent assay kits.

**Results:**

LPS alone significantly increased IL-6 and TNF-α production compared with the control treatment. The treatment of cells with linagliptin at all concentrations significantly inhibited the LPS-stimulated IL-6 and TNF-α production. Meanwhile, IL-1β alone significantly increased IL-6 production compared with the control treatment. No significant difference in IL-6 production was noted between the cells treated with IL-1β and simultaneous treatment with IL-1β and linagliptin.

**Conclusions:**

Linagliptin inhibited LPS-induced inflammation in human monocytic U937 cells.

## Background

The global prevalence of diabetes mellitus continues to increase [1]. Atherosclerosis and inflammation are more common in patients with diabetes than in patients without diabetes, and atherosclerosis progression contributes to inflammation. Therefore, anti-inflammatory therapy is important for the prognosis of patient with diabetes.

Monocytes transform into macrophages in arterial walls and absorb oxidized low-density lipoprotein, forming foam cells via scavenger receptors [2, 3]. They grow and eventually rupture, spilling oxidized materials and promoting atherosclerosis through expansion of the plaque [4, 5]. Therefore, monocytes play a central role in atherosclerosis, and effective control of monocytes may prevent atherosclerosis.

Dipeptidyl peptidase-4 (DPP-4) inhibitors are therapeutic drugs used in patients with diabetes because they activate incretin hormones such as glucagon-like peptide-1. Only the general antidiabetic effects of all nine DPP-4 inhibitors are known. Therefore, it is important to determine the fundamental pharmacological mechanism of each inhibitor to use the appropriate drug for suitable patients. Linagliptin is the only bile-excreted, anti-diabetic oral DPP-4 inhibitor; therefore, its dose reduction is unnecessary [6–8]. Moreover, linagliptin decreased the risk of cardiovascular and cerebrovascular diseases, which are associated with systemic atherosclerosis and related prognostic factors of diabetes [9–11].

We previously reported the in vitro anti-inflammatory effects of linagliptin using human umbilical vein endothelial cells (HUVEC) [12, 13]. On the other hand, the effects of linagliptin on monocytes, which play a central role in arteriosclerosis-related inflammation, are yet to be determined. Moreover, few studies have examined the effects of linagliptin using monocytes. To prevent the progression of atherosclerosis in patients with diabetes, it is important to study the anti-inflammatory effects of linagliptin in monocytes.

Using human monocytic U937 cells, this study aimed to assess the anti-inflammatory effects of linagliptin in human U937 monocytes, which may help establish linagliptin as a key anti-inflammatory drug.

## Methods

### Study materials and cell culture

Linagliptin compounds were provided by Boehringer Ingelheim Pharmaceuticals, Inc. (Ingelheim am Rhein, Rhineland-Palatinate, Germany); ketoprofen and loxoprofen sodium salt dihydrate (Loxo) and interleukin (IL)-1β from Wako Pure Chemical Industries Ltd. (Osaka, Japan); and lipopolysaccharide (LPS) from *Escherichia coli* 055:B5 from Sigma-Aldrich (St. Louis, MO, USA) and phorbor 12-myristate 13-acetate (PMA) from abcam. All chemicals used in this study were of the purest grade available commercially.

Human U937 monocytes (EC85011440) were purchased from the European Collection of Animal Cell Culture UK. All the following operations were carried out under sterile conditions. The cells at densities of 1 × 10^6^ cells/mL were cultured via routine methods in Roswell Park Memorial Institute medium (RPMI 1640) (Wako Pure Chemical Industries Ltd., Osaka, Japan) containing L-glutamine and phenol red with 10% of fetal bovine serum (FBS) (Biosera, NUAILLE, France), 100 nM PMA, 1% of antibiotic-antimycotic solution from gibco by Life Technologies (Carlsbad, CA) at 37 °C, and 5% CO_2_ for 48 h for differentiation into macrophages. The cells were then subsequently used in the experiment.

### Drug treatment

All drug treatments were carried out under aseptic conditions in medium lacking FBS, bovine brain extracts, and hydrocortisone to prevent an increase in the spectrophotometric absorbance through binding with coating antibodies.

The media were replaced, and the cells were pretreated with 1, 5, 10, 50, and 100 nM linagliptin; 50 nM ketoprofen; or 17 μM Loxo for 1 h or left untreated. The media were then replaced again, and the cells were treated with 1 μg/mL LPS or 10 nM IL-1β only, in combination with 1, 5, 10, 50, and 100 nM linagliptin; 50 nM ketoprofen; or 17 μM Loxo or left untreated. An LPS and IL-1β concentration of 1 μg/mL and 10 nM, respectively, was sufficient to induce inflammation in U937 cells. The extracted media were used to measure IL-6 levels 5 and 24 h after drug treatment and tumor necrosis factor (TNF)-α 24 h after drug treatment.

### Measurement of IL-6 levels in the supernatant

We measured IL-6 levels using Human IL-6 Quantikine enzyme-linked immunosorbent assay (ELISA) kits (Bio-Techne-R&D Systems Inc., Minneapolis, USA). This assay employs the quantitative sandwich enzyme immunoassay technique. A monoclonal antibody specific for human IL-6 has been pre-coated onto a microplate. Standards and samples were pipetted into the wells, and any IL-6 present is bound by the immobilized antibody. After washing away any unbound substances, an enzyme-linked polyclonal antibody specific for human IL-6 is added to the wells. Following a wash to remove any unbound antibody-enzyme reagent, a substrate solution is added to the wells, and color developed in proportion to the amount of IL-6 bound in the initial step. The color development was stopped, and the intensity of the color was measured. The absorbance was determined at 450 nm using Spectra Max 340 pc (Molecular Devices Co., CA, USA). The results of IL-6 measurements are shown according to the amount of protein in 1 mL of medium.

### Measurement of TNF-α levels in the supernatant

We measured TNF-α levels using Human TNF-α Quantikine High Sensitivity ELISA kits (Bio-Techne-R&D Systems Inc., Minneapolis, USA). This assay employs the quantitative sandwich enzyme immunoassay technique. A monoclonal antibody specific for human TNF-α has been pre-coated onto a microplate. Standards and samples were pipetted into the wells, and any TNF-α present was bound by the immobilized antibody. After washing away any unbound substances, a biotinylated polyclonal antibody specific for human TNF-α was added to the wells. Following a wash to remove any unbound antibody-biotin reagent, an enzyme-linked streptavidin was added to the wells. After washing away any unbound streptavidin-enzyme reagent, a substrate solution was added to the wells, and color developed in proportion to the amount of TNF-α bound in the initial step. The color development was stopped, and the intensity of the color was measured. The absorbance was determined at 450 nm using the Spectra Max 340 pc (Molecular Devices Co., CA, USA). The results of TNF-α measurements are shown according to the amount of protein in 1 mL of medium.

### Statistical analysis

The results are expressed as mean ± standard error of the mean. Various treatment effects were compared with untreated control cells and LPS- or IL-1β-only treated cells using one-way analysis of variance and Dunnett post hoc analysis. A one-sided *P* value of < 0.05 was considered statistically significant.

## Results

### Effects of linagliptin treatment on LPS-induced IL-6 production after 24 h

**Fig. 1:**
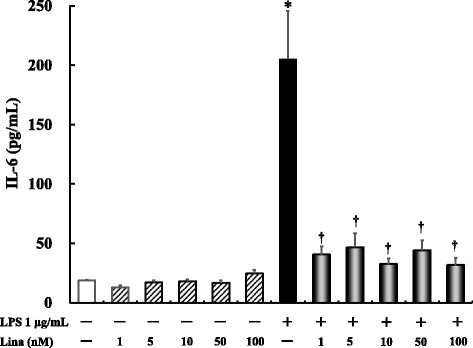
Effects of linagliptin treatment on lipopolysaccharide (LPS)-induced interleukin (IL)-6 production after 24 h. Human U937 monocytes were treated with LPS and/or linagliptin. IL-6 levels in the supernatants were determined via enzyme-linked immunosorbent assay (ELISA) after 24 h of treatment. *P < 0.0001 vs. control; †*P* < 0.0001 vs. LPS 1 μg/mL. Lina, linagliptin

No significant difference in IL-6 production was noted between linagliptin-treated cells at all concentrations and the untreated controls. Further, LPS alone significantly increased IL-6 production compared with the control treatment (*P* ≤ 0.0001). Linagliptin treatment at all concentrations significantly inhibited the LPS-stimulated IL-6 production (*P* ≤ 0.0001) (Fig. 1).

### Effects of linagliptin, ketoprofen, and Loxo treatment on LPS-induced IL-6 production after 24 h

**Fig. 2:**
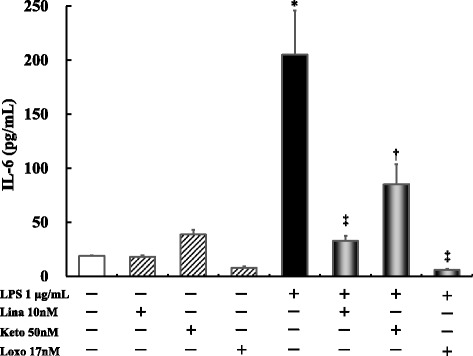
Effects of linagliptin, ketoprofen, or Loxo treatment on LPS-induced IL-6 production after 24 h. Human U937 monocytes were treated with LPS and/or linagliptin, ketoprofen, or Loxo. IL-6 levels in the supernatants were determined via ELISA after 24 h of treatment. *P < 0.0001 vs. control; †*P* < 0.001 vs. LPS 1 μg/mL; ‡*P* < 0.0001 vs. LPS 1 μg/mL. Lina, linagliptin; Keto, ketoprofen

For these experiments, we chose the maximum blood concentration achieved with a single administration of the drug in healthy volunteers. Accordingly, 10 nM linagliptin, 50 nM ketoprofen, and 17 μM Loxo were used. No significant difference in IL-6 production was noted between linagliptin-, ketoprofen-, and Loxo-treated cells and the untreated controls. Further, ketoprofen (*P* ≤ 0.001) and Loxo (*P* ≤ 0.0001) inhibited LPS-stimulated IL-6 production to the same extent as linagliptin (*P* ≤ 0.0001) (Fig. 2).

### Effects of linagliptin treatment on LPS-induced TNF-α production after 24 h

**Fig. 3:**
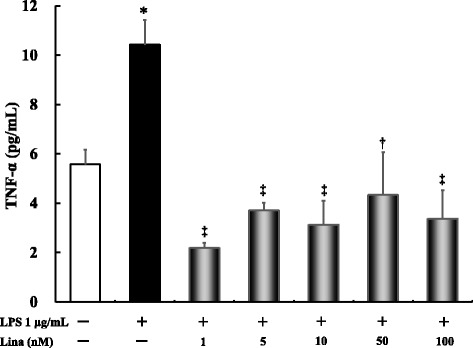
Effects of linagliptin treatment on LPS-induced tumor necrosis factor (TNF)-α production after 24 h. Human U937 monocytes were treated with LPS and/or linagliptin. TNF-α levels in the supernatants were determined via ELISA after 24 h of treatment. *P < 0.001 vs. control; †*P* < 0.01 vs. LPS 1 μg/mL; ‡*P* < 0.0001 vs. LPS 1 μg/mL. Lina, linagliptin

LPS alone significantly increased TNF-α production compared with the control treatment (*P* ≤ 0.001). Linagliptin treatment at all concentrations significantly inhibited the LPS-stimulated TNF-α production (*P* ≤ 0.001) (Fig. 3).

### Effects of linagliptin treatment on LPS-induced IL-6 production after 5 h

**Fig. 4:**
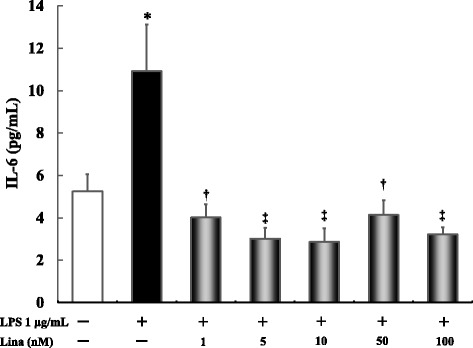
Effects of linagliptin treatment on LPS-induced interleukin IL-6 production after 5 h. Human U937 monocytes were treated with LPS and/or linagliptin. IL-6 levels in the supernatants were determined via ELISA after 5 h of treatment. *P < 0.001 vs. control; †*P* < 0.001 vs. LPS 1 μg/mL; ‡*P* < 0.0001 vs. LPS 1 μg/mL. Lina, linagliptin

LPS alone significantly increased IL-6 production compared with the control treatment (*P* ≤ 0.001). Linagliptin treatment at all concentrations significantly inhibited the LPS-stimulated IL-6 production (*P* ≤ 0.001) (Fig. 4).

### Effects of linagliptin treatment on IL-1β-induced IL-6 production after 24 h

**Fig. 5:**
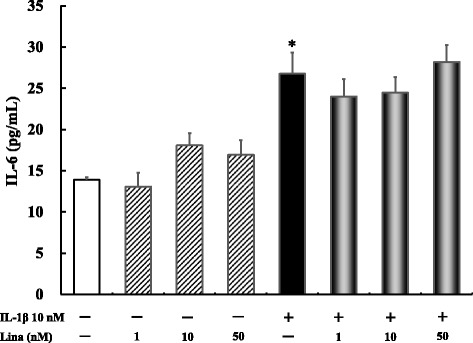
Effects of linagliptin treatment on IL-1β-induced IL-6 production after 24 h. Human U937 monocytes were treated with LPS and/or linagliptin. IL-6 levels in the supernatants were determined via ELISA after 24 h of treatment. *P < 0.0001 vs. control. Lina, linagliptin

For these experiments, 1, 10, and 50 nM linagliptin were used. No significant difference in IL-6 production was noted between linagliptin-treated cells at all concentrations and the untreated controls. Further, IL-1β alone significantly increased IL-6 production compared with the control treatment (*P* ≤ 0.0001). However, no significant difference in IL-6 production was noted between the IL-1β-treated group and simultaneous treatment with IL-1β and linagliptin at all concentrations (Fig. 5).

## Discussion

In this study, pretreatment of human U937 monocytes with linagliptin significantly inhibited LPS-induced IL-6 and TNF-α production, two major inflammatory markers. To date, only two studies have reported on the in vitro anti-inflammatory effects of DPP-4 inhibitors other than linagliptin [14, 15]. Ta et al. found that alogliptin suppresses toll-like receptor (TLR) 4-mediated extracellular signal-regulated kinase activation and matrix metalloproteinase expression in human U937 monocytes [14]. Hiromura et al. found that teneligliptin suppressed LPS-induced expression of pro-inflammatory cytokines in human U937 monocytes [15]. However, there are no reports on the anti-inflammatory effects of linagliptin. To the best of our knowledge, this study is the first to investigate the anti-inflammatory effects of linagliptin in vitro using Human U937 monocytes.

Monocytes play a central role in atherosclerosis, and effective control of monocytes may prevent atherosclerosis [2–5]. Moreover, DPP-4 is expressed as CD26 on various cell membranes, where it acts as an inflammatory mediator; therefore, inflammation can be modulated by DPP-4 inhibition [16–18]. Among the various cells, leukocytes are believed to be the most related to inflammation. Therefore, we used monocytes, which are one of the leukocytes, in this study. In the previous report, we have already mentioned an anti-inflammatory effect of linagliptin through NF-kappa B/p38 phosphorylation in HUVECs [12]. We changed cell line from HUVECs to monocyte in this experiment. Among the various cells, leukocytes are believed to be the most related to inflammation by DPP-4 inhibition. Therefore, we speculated that the anti-inflammatory effects in this experiment using U937 were higher than those using HUVECs. In fact, this experiment using U937 showed stronger anti-inflammatory effect than using HUVECs. The mechanism of anti-inflammation by linagliptin may be due to strong inhibition of CD26 and several other factors.

Following single administration of 5 mg linagliptin, the maximum blood concentration of linagliptin was 7.32 and 16.7 nM in healthy volunteers and patients with diabetes with normal renal function, respectively (Tradjenta® (linagliptin), Boehringer Ingelheim Pharmaceuticals, Inc.). This increased to 22.6 nM after repeated administration of 5 mg linagliptin in patients with diabetes and a creatinine clearance of ≤ 30 mL/min (Tradjenta® (linagliptin), Boehringer Ingelheim Pharmaceuticals, Inc.) [19]. We assumed that the maximum blood concentration is over 22.6 nM after repeated administration of 5 mg linagliptin in patients with end-stage renal disease. Therefore, we decided to use doses from 1 to 100 nM linagliptin in patients whose blood concentration of linagliptin are equivalent to that of actual clinical treatment.

We also investigated the strength and mechanism of the anti-inflammatory effects of linagliptin by measuring the IL-6 production after linagliptin, ketoprofen, and Loxo treatment. The working concentrations of all three drugs were selected based on their maximum blood concentrations after single administration in healthy volunteers. Linagliptin, ketoprofen, and Loxo, the latter two being representative anti-inflammatory drugs, significantly inhibited LPS-stimulated IL-6 production to a similar extent.

IL-6 production also changes depending on the difference in extraction time from LPS or linagliptin administration. In our previous study using HUVEC, the extraction time was only 5 h [12, 13]. However, the anti-inflammatory effect by linagliptin was observed in both the extraction time of 5 and 24 h in this experiment. These results indicate that the anti-inflammatory effect of linagliptin could be sustained for a long time.

It is important to mimic the experimental models with agents other than LPS. Therefore, we also investigated the anti-inflammatory effects of linagliptin following stimulation of human U937 monocytes with IL-1β. Linagliptin treatment did not inhibit IL-1β-stimulated IL-6 production. In our previous study using HUVEC, linagliptin prevented LPS- and indoxyl sulfate-induced IL-6 production [12, 13]. LPS binds to TLR 4 receptor or advanced glycation end product and induces pro-inflammation, whereas IL-1β binds to the IL-1β receptor. Different pro-inflammatory agents have different acting receptors, and this might have changed the anti-inflammatory effects of linagliptin.

One of the limitations of this study is that the anti-inflammatory effect of linagliptin is not dose-dependent. In order to elucidate the dose dependence of the anti-inflammatory effect, it is necessary to study the anti-inflammatory effect with below 1 nM of linagliptin concentration in the future. The other limitation of this study is that IL-1β-induced IL-6 was not suppressed by linagliptin. In general, the IL-1 receptor (IL-1R) and LPS receptor TLR4 are defined as a family of proteins, and their several common and individual downstream signaling have been reported (MyD88, mitogen-activated protein kinase). However, in recent years, receptor for advanced glycation end products (RAGE) is reported as for receptor of LPS in addition to TLR4 [20]. After LPS binds to RAGE, NF-κB, IL-6, and TNF-α are synthesized. The suppression of LPS-induced IL-6 and non-suppression of IL-1β-induced IL-6 might be due to the difference in involved in such receptors (RAGE, IL-1 receptor, TLR4) by linagliptin. It is necessary to examine differences in involved in receptor by linagliptin to elucidate the anti-inflammatory mechanism by linagliptin.

## Conclusion

We confirmed that linagliptin inhibited LPS-induced inflammation in human U937 monocytes. Our results highlight the clinical significance of linagliptin in the treatment of atherosclerosis and inflammation in patients with diabetes.

## Abbreviations

DPP-4: Dipeptidyl peptidase-4
ELISA: Enzyme-linked immunosorbent assay
FBS: Fetal bovine serum
HUVEC: Human umbilical vein endothelial cells
IL: Interleukin
Loxo: Loxoprofen sodium salt dihydrate
LPS: Lipopolysaccharide
PMA: Phorbol myristate acetate
TLR: Toll-like receptor
TNF: Tumor necrosis factor
